# Molecular Mechanisms and Nutritional Modulation in Sarcopenia: A Narrative Review

**DOI:** 10.3390/nu18071161

**Published:** 2026-04-05

**Authors:** Hui San Chin, Ling Liu, Pei-Ju Liao, Alexandra L. R. M. Wee, Xiu-Yi Kwek, Bin Tean Teh, Frederick H. Koh

**Affiliations:** 1 Research Office, Sengkang General Hospital, Singapore 544886, Singapore; liu.ling@skh.com.sg (L.L.); liao.pei.ju@skh.com.sg (P.-J.L.); alexandra.louise.w.r@skh.com.sg (A.L.R.M.W.); kwek.xiu.yi@skh.com.sg (X.-Y.K.); 2 SingHealth Duke-NUS Muscle Health Program, SingHealth, Singapore 168582, Singapore; teh.bin.tean@singhealth.com.sg; 3 Surgery Academic Clinical Programme, Duke-NUS Medical School, National University of Singapore, Singapore 169857, Singapore; 4 Genome Institute of Singapore, Agency for Science, Technology and Research (A*STAR), Singapore 138672, Singapore; 5 Laboratory of Cancer Epigenome, Division of Medical Science, National Cancer Center Singapore, Singapore 168583, Singapore; 6 Institute of Molecular and Cell Biology, Agency for Science, Technology and Research (A*STAR), Singapore 138673, Singapore; 7 Department of General Surgery, Colorectal Service, Sengkang General Hospital, Singapore 544886, Singapore

**Keywords:** sarcopenia, muscle regeneration, muscle health, nutritional interventions

## Abstract

Sarcopenia is a progressive and multifactorial muscle disorder associated with diminished strength, reduced functional capacity, and increased risk of adverse health outcomes including frailty, falls, and mortality. Despite its clinical burden, the molecular pathogenesis of sarcopenia remains poorly understood, which hinders the development of precise therapeutic strategies. This review examines emerging evidence linking anabolic resistance, mitochondrial dysfunction, neuromuscular instability, and chronic inflammation to impaired regeneration and disrupted proteostasis. While nutritional interventions such as high-quality protein, leucine metabolites, and vitamin D supplementation preserve lean mass, they fail to consistently restore function independently. Although exercise remains the cornerstone therapy, its benefits are often constrained in patients with multimorbidity or reduced mobility. Given the biological heterogeneity of sarcopenia, there is a need to shift from generic supportive care to stratified, mechanism-based therapy. Emerging omics technologies including transcriptomic, proteomic, and metabolic profiling offer a promising avenue to define molecular endotypes. This will guide the development of precision-based management strategies.

## 1. Introduction

Sarcopenia, defined as the progressive loss of skeletal muscle mass and function, is widely acknowledged as a critical determinant of health outcomes in aging populations and patients with chronic disease. However, a universal consensus on clinical definitions and diagnostic criteria remains poorly defined [1]. Furthermore, the clinical evaluation of appendicular skeletal muscle remains an evolving area, with a need for accessible tools that can accurately capture changes across muscle mass, quality, and functional capacity [2]. Once regarded as an inevitable consequence of aging, sarcopenia is now understood as a complex, multifactorial syndrome with systemic consequences that extend far beyond locomotion [3,4]. Individuals with sarcopenia face a significantly elevated risk of falls, fractures, cognitive impairment, and functional dependency, leading to increased hospitalization rates, prolonged lengths of stay, and greater healthcare costs [5,6,7]. Moreover, low muscle strength is an independent predictor of all-cause mortality and frequently co-exists with cardiometabolic disease, osteoporosis, and cognitive impairment. This highlights the central role of skeletal muscle in whole-body homeostasis. Additionally, skeletal muscle is the body’s largest endocrine organ and a primary regulator of glucose disposal, amino acid availability, and energy balance [3]. When muscle health declines, it disrupts the tightly integrated metabolic and immune systems, adding to the acceleration of multimorbidity.

Despite the growing awareness, the pathophysiology of sarcopenia remains incompletely defined, in part because multiple biological processes are often described in isolation rather than as components of an integrated system. Key mechanisms, including anabolic resistance, mitochondrial dysfunction, and neuromuscular instability, are highly interconnected and often occur concurrently during aging and disease. Broadly, anabolic resistance limits protein synthesis despite adequate nutrient and mechanical stimuli, mitochondrial dysfunction reduces energy availability and increases oxidative stress, and neuromuscular junction (NMJ) degeneration impairs motor unit recruitment and force generation [8,9,10,11]. Chronic inflammation and metabolic stress further amplify dysfunction across these domains [12]. This integrated framework provides a more coherent biological basis for understanding disease progression and is explored in greater mechanistic detail in the following sections. Collectively, these molecular disruptions impair muscle strength, function and physical performance, which often decline disproportionately relative to muscle mass.

A further challenge lies in the clinical and biological heterogeneity of sarcopenia. While often framed as an age-related condition, similar phenotypes arise in chronic disease, physical inactivity and disuse, each driven by distinct but overlapping mechanisms. Age-related sarcopenia is characterized by gradual motor unit loss, mitochondrial dysfunction, and hormonal decline [13]. In chronic disease, catabolic inflammation, hypoxia, and insulin resistance accelerate functional decline even in the presence of preserved muscle mass [3]. Inactivity-related sarcopenia, seen in hospitalization or bedrest, leads to rapid functional deterioration due to neuromuscular deconditioning [14]. Sarcopenic obesity further adds to the complexity, where fat infiltration and inflammatory signaling undermine function despite normal or elevated body mass [15]. While these subtypes reflect clinical diversity, this review focuses on age-related sarcopenia, where nutritional and mechanical interventions remain most actionable.

While international frameworks such as the Asian Working Group for Sarcopenia (AWGS), European Working Group on Sarcopenia in Older People (EWGSOP2), and the Global Leadership Initiative in Sarcopenia (GLIS) identify nutrition as a foundational component or care, nutritional strategies often fail to restore function when applied in isolation [16,17,18,19]. This disconnect between gains in muscle mass and limited improvement in strength or function highlights the importance of targeting muscle quality and function, rather than muscle mass alone. The biological heterogeneity of sarcopenia, driven by distinct defects in mitochondrial quality control, neuromuscular stability, and inflammatory signaling, suggests that the current “one-size-fits-all” approaches are inadequate. Emerging multi-omics frameworks are beginning to better characterize this heterogeneity by identifying distinct mechanistic pathways involved in sarcopenia [20,21,22]. This has led to increasing interest in stratified and mechanism-based approaches, where interventions are aligned with dominant biological drivers. However, clinical implementation remains limited and requires clearer integration between molecular insights and functional outcomes.

Current nutritional and exercise-based strategies remain largely non-specific and do not account for this heterogeneity [23]. Nutritional interventions can preserve or modestly increase lean mass, but their effects on strength and function are inconsistent. While resistance exercise remains the cornerstone of therapy, its implementation is often constrained in individuals with frailty, multimorbidity, or limited mobility, restricting its applicability [24]. Even combined multimodal interventions yield variable responses, likely reflecting differences in underlying molecular drivers and patient capacity. Stratifying patients based on these molecular drivers may enable more targeted and effective interventions.

This review bridges the gap between mechanistic biology and translational care. Advances in muscle regeneration are summarized, with emphasis on how core pathological drivers, including mitochondrial dysfunction and stem cell dysregulation, limit the efficacy of current interventions. These disruptions converge on key processes such as reduced neuromuscular integrity, impaired energy metabolism, and blunted anabolic signaling, ultimately contributing to declines in muscle strength and function. The role of multimodal prehabilitation and emerging multi-omics approaches for patient stratification is also discussed. Ultimately, sarcopenia management is increasingly moving toward stratified, mechanism-based precision medicine. This narrative review was conducted through a targeted, non-systematic appraisal of the literature, focusing on key mechanistic and clinical studies in sarcopenia, muscle biology, and nutritional interventions. Relevant publications were identified using major databases and prioritized based on the quality of research and recency. As a narrative synthesis, formal systematic review methodology and risk-of-bias assessment, were not applied.

## 2. Mechanisms Underlying Sarcopenia and Muscle Regeneration

**Figure 1:**
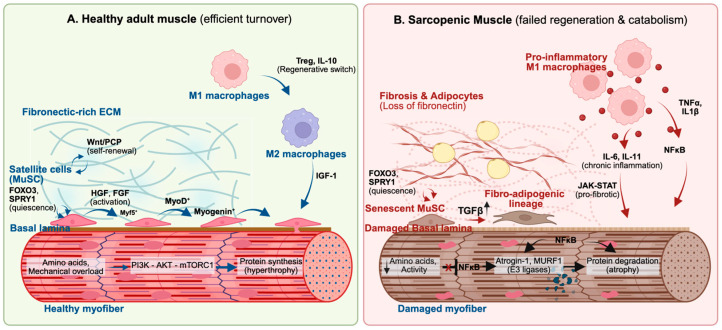
Molecular drivers of healthy muscle regeneration versus sarcopenic muscle. (**A**) Healthy adult muscle: efficient repair is maintained by satellite stem cell (MuSC) renewal and Treg-driven immune regenerative switch. Anabolic signals (amino acids, insulin, mechanical overload) promote protein synthesis through the PI3K–AKT–mTORC1 pathway. (**B**) Sarcopenic muscle: loss of fibronectin-rich ECM, coupled with fibrotic changes and adipocyte infiltration disrupts MuSC quiescence and self-renewal, leading to exhaustion, or stress-induced senescent state. Elevated TGFβ diverts progenitors to maladaptive fibro-adipogenic lineages, while chronic inflammation blocks regeneration and upregulates catabolic processes.

Sarcopenia is fundamentally a failure of the molecular systems maintaining skeletal muscle. While clinically defined by the loss of mass and function, it arises from the breakdown of spatiotemporal coordination between resident stem cells, immune populations, and the extracellular matrix (ECM). Aging and chronic inflammation disrupt these processes, shifting tissue away from efficient turnover, toward catabolism (Figure 1).

### 2.1. Disruption of Satellite Cell Dynamics and Quiescence

An important contributor to sarcopenia is the dysfunction of satellite cells (MuSC), the resident muscle stem cell located beneath the basal lamina [25]. In healthy muscle, FOXO3 and SPRY1 maintain quiescence, while niche-derived signals like HGF and FGF trigger activation, prompting MuSC to proliferate and differentiate via the sequential expression of Myf5, MyoD, and Myogenin (MyoG). These regulatory pathways are largely defined in murine models, where FOXO3 deletion disrupts quiescence and SPRY1 loss depletes the MuSC pool following repeated injury [26,27].

Fibronectin, via integrin-β1 promotes MuSC self-renewal through the non-canonical Wnt/Planar Cell Polarity (PCP) pathway [28]. Disruption of this axis impairs regenerative capacity and limits progenitor replenishment in preclinical models [28]. Myogenic identity declines as senescence markers accumulate and elevated TGF-β diverts differentiation toward maladaptive fibro-adipogenic lineages [29]. Importantly, human transcriptomic studies confirm age-related alterations in FN1, ITGB1, and SPRY1, supporting translational relevance [30]. However, causality remains nuanced. Experimental depletion studies suggest that MuSC loss alone does not accelerate sarcopenia under sedentary conditions, indicating that MuSC dysfunction is insufficient to drive age-related atrophy [31]. Rather, MuSCs primarily modulate regenerative capacity within a permissive niche environment.

In humans, reductions in satellite cell number, particularly in type II fibers, are consistently observed with aging and associated with fiber atrophy and strength decline [32,33]. Functional links are further supported by training studies showing that satellite cell expansion accompanies gains in muscle strength and fiber size [34,35]. MuSC dysfunction may be a context-dependent contributor that amplifies, rather than an initiator of sarcopenia.

### 2.2. Failure of the Immune Regenerative Switch

Muscle regeneration relies on a “regenerative switch” where regulatory T cells (Tregs) and IL-10 drive macrophages from the pro-inflammatory (M1) to a pro-regenerative (M2) phenotype, essential for IGF-1 secretion during myogenesis [36,37,38,39,40]. This transition is an active, signal-dependent process regulated by AMPKα1, HGF/c-Met, and phagocytic pathways, rather than a passive temporal shift [41,42,43]. In sarcopenia, this precise coordination is disrupted, with dysregulations to both the initiation and resolution of inflammation, resulting in persistent or mistimed macrophage activation [44]. 

Chronic inflammatory signaling further destabilizes this switch. Sustained IL-6 and IL-11 activate JAK-STAT3, suppressing myogenic differentiation and promoting pro-fibrotic stromal behavior [45,46]. Secondly, TNFα and IL-1β drive canonical NFκB activity that upregulates E3 ubiquitin ligases Atrogin-1 and MuRF1 while blunting anabolic IGF–IRF–Akt signaling [37,47]. Interferon signaling amplifies this network, where IFNγ-JAK/STAT1 and NFκB converge to suppress MyoD and myotube formation, reinforcing a non-regenerative niche [48,49]. Clinically, circulating IL-6, TNFα, and C-reactive protein (CRP) correlate with reduced muscle strength and physical performance, linking immune dysregulation directly to functional decline [50,51]. Collectively, these findings suggest that dysregulation lies not in a single pathway, but in the loss of temporal coordination across inflammatory networks.

### 2.3. Anabolic Resistance and Mitochondrial Decline

In sarcopenia, muscle loss also reflects impaired proteostasis driven by dysfunction across the IGF-1–IRS-1–PI3K–Akt–mTORC1 axis [52,53]. In sarcopenia, this axis is blunted, despite preserved or elevated basal signaling, a paradox that underpins anabolic resistance [54,55]. Mechanistically, chronic mTORC1 hyperactivation promotes S6K1-mediated inhibitory phosphorylation and degradation of IRS-1, uncoupling upstream signaling from downstream protein synthesis [56]. This is compounded by reduced amino acid transporter induction and impaired translational efficiency, limiting myofibrillar protein renewal despite adequate nutrient availability [57].

Chronic inflammation and oxidative stress further suppress anabolic signaling through the NFκB, JNK, and p38 MAPK pathways, promoting inhibitory IRS-1 phosphorylation and catabolic activation [58,59]. Concurrently, FOXO3 and NFκB upregulate MuRF1 and Atrogin-1, driving preferential degradation of fast-twitch contractile proteins and reducing specific force and function [60,61]. Underlying these defects, mitochondrial dysfunction imposes energetic and redox constraints. Declining NAD+ levels and reduced PGC-1α impair oxidative phosphorylation, while defective mitophagy increases ROS and further suppresses anabolic signaling [62]. PGC-1α, a key regulator of mitochondrial biogenesis, is reduced in aging human muscle and is inducible with exercise. However, its role as a therapeutic target in sarcopenia remains unproven in a clinical setting [63,64]. Mitochondrial respiratory capacity correlates more strongly with strength and physical performance than muscle mass, emphasizing the mass and function dissociation [65,66]. Human studies link mitochondrial capacity to endurance and fatigability, and walking performance supports its translational relevance [67,68,69], although the direct relationship to maximal strength is less clearly established [69]. Collectively, these findings position anabolic resistance as a systems-level signaling defect integrating impaired signaling, mitochondrial dysfunction, and catabolic dominance, explaining why strength and function decline disproportionately to muscle mass in aging.

### 2.4. Niche Changes: Fibrosis and Neuromuscular Junction (NMJ) Instability

In sarcopenia, the stromal and ECM niche undergo pathological remodeling that actively hinder repair. TGFβ driven SMAD2/SMAD3 signaling promotes collagen deposition, stiffening the microenvironment and impairing YAP/TAZ-mediated mechanotransduction [70]. This fibrotic remodeling impairs force transmission, reducing specific force independent of muscle mass and reinforcing the dissociation between muscle quantity and function [71]. Concurrently, the NMJ becomes impaired and undergoes structural degradation, even before overt muscle loss becomes evident. Age-related NMJ instability is driven by the reduced expression of MUSK, LRP4, and AGRIN, which are necessary for acetylcholine receptor (AChR) clustering and synaptic stability. Both experimental and human studies demonstrate that NMJ degeneration precedes myofiber atrophy and is detectable in pre-sarcopenic individuals, supporting its role as an early contributor to functional decline [72].

This degradation is exacerbated by the age-related upregulation of neurotrypsin, cleaving agrin into 22 kDa C-terminal fragment (CAF). Elevated circulating CAF levels are associated with reduced physical function and are emerging as circulating biomarkers for NMJ degeneration in sarcopenia. Histological analyses further demonstrate denervation muscle fibers in older adults, identified by the re-expression of neural cell adhesion molecule (NCAM) and neonatal myosin heavy chain (MHCn). These markers are strongly associated with reduced muscle strength and physical performance and may predict functional capacity more robustly than muscle mass alone [73]. Dysregulated mTORC1 activity impairs AChR turnover, while decreased PGC-1α and mitochondrial dysfunction at the synapse promote oxidative stress and transmission failure. Together, these neuromuscular and fibrotic alterations accelerate the loss of fast-twitch fibers and functional capacity in aging muscle [74,75,76].

These processes are highly interconnected, with MuSC dysfunction, immune dysregulation, mitochondrial dysfunction, and neuromuscular instability converging to impair muscle quality and functional capacity. While many mechanisms are derived from preclinical models, their translation to human disease remains variable, with clinical data highlighting context-dependent effects influenced by aging, comorbidity, and physical inactivity. This disconnect highlights the need to interpret mechanistic findings within an integrated physiological and functional framework.

### 2.5. Limitations of Preclinical Models

Much of our mechanistic understanding of sarcopenia is derived from rodent models and cell lines. However, this knowledge has frequently failed to translate into effective clinical therapeutics [77]. This translational gap stems from biological disparities between species. Not only do rodent models have a distinct muscle fiber composition than humans, but their short lifespan also precludes the decades-long accumulation of environmental stressors that characterize human aging. While most preclinical studies often emphasize muscle size or mass, human sarcopenia is defined by functional decline. This focus on mass-centric data obscures the clinical reality that hypertrophy does not guarantee functional recovery.

These limitations are not exhaustive and include factors such as co-morbidities, including obesity, diabetes, and cognitive decline, age-related alterations in immune system functions, and differences in pharmacokinetics and pharmacodynamics. These variables modulate inflammatory signaling, metabolic homeostasis, and anabolic responsiveness, thereby contributing to the frequent disconnect between mechanistic efficacy in preclinical models and functional outcomes observed in human sarcopenia.

## 3. Nutrition and Sarcopenia

Skeletal muscle maintenance relies on the coordinated regulation of protein turnover, mitochondrial energetics, and inflammatory signaling. Nutrition modulates each of these domains and is recognized across international consensus frameworks as a foundational component of sarcopenia management [78]. Nutrients influence mTORC1 activity, autophagy, mitochondrial respiration, and endocrine tone. However, the clinical effectiveness of nutrition intervention is highly context-dependent and relies heavily on baseline nutritional status, comorbidity burden, and the presence of concurrent mechanical loading.

### 3.1. Protein and Amino Acids

Protein remains the primary nutritional driver of muscle anabolism. To overcome age-related anabolic resistance, older adults require higher protein intakes than younger adults. Current guidelines recommend 1.0–1.2 g/kg/day for healthier older adults, and ≥1.2 g/kg/day for individuals with sarcopenia, frailty, or chronic disease (Table 1). Adults engaged in resistance training may benefit from ≥1.6 g/kg/day.

Beyond total quantity, amino acid composition is critical. Leucine acts as a potent regulator by stimulating the mTORC1 pathway to drive protein synthesis. Leucine-rich sources like whey protein are particularly effective at enhancing this response, supporting post-exercise recovery. Furthermore, the leucine metabolite β-hydroxy-β-methylbutyrate (HMB) confers distinct, complementary benefits [79,80,81]. While leucine primarily drives synthesis, HMB functions by reducing proteolysis and modulating inflammatory signaling, preserving muscle integrity in catabolic states [82]. However, this anabolic mechanism is complicated in sarcopenia. Under healthy conditions, supplemental leucine or branched-chain amino acids (BCAAs) trigger a productive rise in mTORC1-driven protein synthesis. In contrast, multi-omics studies reveal that sarcopenia is characterized by disrupted mitochondrial BCAA catabolism [83]. This leads to an intracellular accumulation of amino acids, a paradoxical state of chronic mTORC1 activation that becomes maladaptive rather than anabolic. Experimental models demonstrate that sustained mTORC1 signaling promotes oxidative stress, mitochondrial dysfunction, and preferential loss of fast glycolytic fibers, thereby impairing muscle quality and functional capacity despite preserved or elevated anabolic signaling [56].

### 3.2. Vitamins and Omega-3 Fatty Acids

Micronutrients support muscle maintenance, shaping the cellular environment required for anabolic response. Vitamin D supports protein synthesis, mitochondria respiration, and calcium handling [84,85]. Antioxidants (Vitamin C, vitamin E, selenium, and magnesium) temper oxidative stress, support mitochondrial integrity and limit redox-driven proteolysis. Similarly, in preclinical models, omega-3 fatty acids exert anti-inflammatory effects and modulate several pathways including intramuscular lipid metabolism, mTORC1 signaling, improved mitochondrial function, reduced proteolysis, and enhanced amino-acid transport [86,87,88]. Despite these mechanistic effects, clinical outcomes remain inconsistent, with several meta-analyses reporting modest or non-clinically meaningful improvements in muscle mass or physical performance [89,90].

### 3.3. Creatine

Creatine remains one the most consistently supported supplements for older adults, acting at the intersection of energetics and anabolism. Its efficacy stems from three mechanisms [91]. First, it replenishes intramuscular phosphocreatine (PCr) stores to regenerate ATP, enhancing training capacity and augments strength outcomes. Second, as an osmolyte, creatine triggers cellular hydration, triggering a distinct anabolic signal that activates mTORC1 and satellite-cell activity. Finally, it possesses antioxidant properties, improving mitochondrial ATP flux and attenuating pro-inflammatory cytokines. Clinically, creatine consistently augments strength and functional performance when combined with resistance training [92]. Recent meta-analyses further demonstrate that creatine supplementation produces significantly greater improvements in muscle strength and lean tissue mass when administered alongside resistance exercise compared with exercise alone, highlighting the synergistic interaction between energetic support and mechanical loading in aging muscle. In contrast, creatine supplementation without concurrent exercise does not produce significant improvements in muscle function [93].

### 3.4. Emerging Metabolic Regulators

Research is increasingly focusing on metabolites that target cellular quality control. Urolithin A, a gut-derived metabolite, activates PINK1-parkin to drive mitophagy, enhancing mitochondrial gene expression and muscular endurance [94]. Its actions parallel those of spermidine, a polyamine that induces autophagy [95]. Notably, circulating spermidine levels are often depleted in sarcopenic adults [96]. A second class of regulators targets NAD+ metabolism. Precursors such as nicotinamide riboside (NR) and nicotinamide mononucleotide (NMN) restore intracellular NAD+ pools, activating sirtuin-driven mitochondrial biogenesis [97]. Finally, the nitric oxide precursors (L-citrulline and L-arginine) improve perfusion and contractile physiology [81].

### 3.5. Gut-Microbiome

The gut microbiome serves as a key regulator of muscle health. Age-related dysbiosis may reduce short-chain fatty acid (SCFA) production, promoting systemic inflammation [98]. Targeted probiotics (*Lacticaseibacillus paracasei* PS230) and fiber-rich diets may restore microbial balance and have demonstrated modest improvements in lower-limb strength and inflammatory markers [99].

Ultimately, nutrition rarely succeeds in isolation, as summarized across key nutritional strategies and their clinical effects (Table 2). Large-scare meta-analyses demonstrate that protein supplementation alone does not consistently improve muscle strength or physical performance in older adults, despite their modest or context-dependent effects on lean mass [100,101,102]. In contrast, combined interventions integrating resistance training with nutritional support consistently demonstrate superior gains in muscle mass, strength, and functional performance compared with nutrition alone [103,104]. These findings indicate that while nutrition modulates key molecular pathways including mTORC1 signaling, mitochondrial energetics, inflammatory regulation, and neuromuscular integrity, it primarily supports anabolic priming rather than drive functional adaptation. The translation into meaningful gains in muscle strength and function depends on concurrent mechanical loading, highlighting the importance of integrated, multimodal strategies in sarcopenia management.

## 4. Pharmacological Interventions in Sarcopenia

The pharmacological landscape for sarcopenia remains investigational, with no FDA-approved pharmaceutical interventions [23]. This therapeutic gap persists due to challenges in trial design and profound heterogeneity of the older adult population. Key investigational agents and their mechanistic targets are summarized in Table 3.

Historically, research prioritized anabolic agents to counteract age-related hormonal declines. While testosterone, selective androgen receptor modulators (SARMs), and myostatin/activin inhibitors (e.g., bimagrumab) reliably increase lean mass, these structural gains fail to translate into meaningful functional improvements [110,111,112,113]. This persistent “mass-function dissociation” implies that mere structural hypertrophy is insufficient to restore neuromuscular performance.

Emerging strategies aim to mitigate chronic low-grade inflammation (“Inflammaging”) through TNFα modulation or senolytics that clear senescent cells [114,115]. A second, parallel strategy targets the profound mitochondrial dysfunction observed in aging muscle [10]. This area includes NAD+ analogues to correct declining NAD+ levels and mitophagy activators, such as Urolithin A, to clear damaged mitochondria [94,116]. Even repurposed agents with strong mechanistic rationales, such as renin–angiotensin–aldosterone system (RAAS) modulators (e.g., Perindopril, Losartan), demonstrated early promise in preclinical models but did not show consistent benefits in large, randomized trials [117,118].

**Table 3:** Key pharmacological targets for sarcopenia.

Target Pathway	Agent Class	Mode of Action	Efficacy & Limitations
Anabolic pathways	Androgens (growth hormone, like testosterone)	Binds androgen receptors and promotes protein synthesis	Increased in mass & strength. Limitations: adverse effect including cardiovascular risk, polycythemia, androgenic effects [112,119,120].
	SARMs (Enobosarm, MK-0773)	Selectively binds androgen receptors in muscle/bone	Increased in lean mass. Improvement in stair climb. Limitations: No consistent strength gain and liver toxicity [121,122,123,124].
Myostatin pathway	Myostatin inhibitors (Landogrozumab)	Neutralizing myostatin	Increased in muscle mass. Limitations: fails to improve strength and/or function [113].
	Activin II receptor blockers (Bimagrumab)	Neutralizing myostatin and activin A	Increased in muscle mass and strength (phase II trial) with inconsistent functional benefit in larger trials [125,126,127].
Inflammation	Anti-inflammatories (Ibuprofen, Tofacitinib)	Reduces prostaglandin synthesis. JAK-2 inhibition	Linked to lower sarcopenia risk. However, limited randomized clinical trial [128,129].
	Senolytics (Dasatinib, Quercetin)	Induces apoptosis in senescent cells	Improved physical function in preclinical mouse models but lacking in evidence in human [129,130,131].
Mitochondrial health	NAD+ precursors (NR, NMN, trigonelline)	Replenishes intracellular NAD+ pools; activates sirtuins	Inconsistent findings in human clinical trials with mixed improvement in strength and mass [132,133].
	Mitophagy activators (Urolithin A)	Activates PINK1–Parkin mitophagy axis	Increased in muscle endurance. Small RCTs show mitochondrial health. Limitations: modest effects with unproven mass gains [134,135].
	Metabolic modulators (metformin)	Activates AMPK signaling and surpasses inflammation	Being explored in combination therapies while efficacy as monotherapy is unclear [136,137].
Novel/repurposed	RAAS modulators (perindopril, losartan)	ACE inhibitors/ARB, may improve perfusion	Preclinical promise (fibrosis reduction). Limitations: failed to show efficacy in human randomized clinical trials [117,138].
	Troponin activators (CK-2917357)	Sensitizes contractile machinery to calcium	May improve force dependent of mass. Limitations: early-phase development [139,140].

## 5. Exercise, Physical Activity, and Prehabilitation

Exercise remains the cornerstone of sarcopenia management, serving as the only intervention capable of consistently restoring function [141]. Resistance exercise (RE) is the primary anabolic driver. A recent meta-analyses of randomized controlled trials in sarcopnic older adults demonstrated moderate-to-large improvements in muscle strength following resistance training, including handgrip strength, knee extension strength, and gait speed, alongside smaller but significant gains in muscle mass [141,142,143,144]. Moderate-to-vigorous load (70–85% 1 Repetition maximum (RM), 2–3X/week) activates the PI3K–Akt–mTOR axis, enhancing translation initiation and satellite-cell activity [34,118,145,146]. Crucially, sufficient mechanical loading can partially overcome anabolic resistance, restoring the synthetic response even in senescent tissue. This is complemented by aerobic exercise (AE), which engages the AMPK–SIRT1–PGC1α [147,148]. This signaling cascade drives mitochondrial biogenesis, oxidative capacity, and fatigue resistance, creating an optimal metabolic environment to support RE-induced hypertrophy [149]. Exercise also shifts macrophage polarity from pro-inflammatory M1 to M2 pro-regenerative states [150,151].

However, the greatest efficacy arises from multimodal strategies. Meta-analyses consistently confirm that combining RE with nutritional optimization (protein, BCAA/HMB, creatine) yields superior gains in lean mass and mobility compared to monotherapy [152,153,154]. Clinically, this integration is formalized as “prehabilitation”, a structured, multi-domain preparation for major physiological stress, such as oncologic surgery [155,156,157]. Clinical trials demonstrate significant reductions in postoperative complications (~40%) and functional preservation. Despite this success, critical gaps remain regarding standardized dosing and long-term durability. Future efforts must focus on developing “phenotype-matched” prehabilitation, tailoring intervention intensity to specific molecular deficits (e.g., mitochondrial deficits vs. inflammatory subtypes) to optimize outcomes for high-risk patients. Taken together, these findings suggest that although several agents show mechanistic promise or modest effects on lean mass, the field remains limited by inconsistent functional benefit, short trial duration, and uncertainty regarding which biological subgroups are most likely to respond.

## 6. Multi-Omics and Biological Heterogeneity

The transition from symptomatic definitions to mechanistic understanding is critical for addressing sarcopenia. However, omics-based stratification primarily remains a research tool rather than a routine clinical strategy. Most transcriptomic, proteomic, and metabolomic platforms require specialized infrastructure, are costly, and lack standardized thresholds for diagnosis or treatment selection in everyday practice. As such, their near-term value may lie less in immediate bedside deployment and more refining biomarker panels that can later be translated into simpler, clinically accessible assays.

However, the current intervention literature remains limited by substantial heterogeneity in population studies, baseline functional status, exercise design, supplement composition, and outcome selection. Many trials are relatively short in duration, underpowered for clinically meaningful endpoints, and enriched for healthier or more adherent participants, which may overestimate feasibility and response in real-world sarcopenic populations. Importantly, gains in lean mass do not consistently translate into improvements in strength or physical performance. This disconnect highlights the need for future studies that prioritize functional and patient-centered outcomes over structured endpoints alone.

## 7. Conclusions

Sarcopenia is far more than the inevitable decline of aging. It is a complex, multifactorial failure of the biological systems that maintain muscle homeostasis. As this review has outlined, the condition is driven by a convergence of satellite-cell exhaustion, mitochondrial dysfunction, anabolic resistance, and chronic inflammation. While nutrition and exercise remain the cornerstone of management, their efficacy is often limited by the profound heterogeneity of the patient population. While these advances strongly support the conceptual shift toward mechanism-based care, most precision approaches in sarcopenia remain at an early translational stage. At present, exercise, nutritional optimization, and multimodal rehabilitation remain the main clinically actionable strategies, whereas omics-informed stratification should be viewed as a promising future direction that requires further validation before routine implementation. 

## Figures and Tables

**Table 1:** Recommended daily protein intake for older adults and individuals with sarcopenia.

Population Group	Recommended Intake (g/kg/day)	Rationale	Consensus
Healthy older adults	1.0–1.2	Compensate for anabolic resistance and maintain muscle mass	AWGS, ESPEN, PROT-AGE
Older adults with sarcopenia or frailty	≥1.2	Support anabolism and counter catabolic burden	AWGS, ESPEN, GLIS
Adults with chronic disease	1.2–1.5	Address increased metabolic demands and protein turnover	ESPEN
Adults engaging in resistance training	≥1.6	Enhance adaptive hypertrophy and strength gains	PROT-AGE, International sports nutrition

**Table 2:** Representative evidence for key nutritional supplements.

Supplement	Mode of Action	Clinical Findings
Whey protein (leucine rich)	Stimulates mTORC1, boosts amino acid signaling	Significant gain in lean mass (+0.5–1.0 kg); Functional gains only with exercise [105].
HMB (3 g/day)	Supports anabolism, reduces catabolism and lower inflammation	Significant gain in lean mass (0.3–0.7 kg); strength gain is inconsistent [106]; limited as monotherapy without exercise; IMAT shown reduction in selected studies [107].
BCAA	Provide anabolic substrates; modulate mitochondrial signals	Functional improvements only with exercise; minimal effect on lean mass [108].
Vitamin D	Enhances oxidative metabolism and calcium-dependent function	Benefits limited to deficient individuals; no-significant effects when replete [84].
Omega-3 fatty acids	Anti-inflammatory and may improve muscle quality	Effects on muscle mass are inconsistent and generally small [89].
Creatine (3–5 g/day)	Increases energy availability for contraction and training	Consistent improvements in strength and performance with exercise (*p* < 0.05); No significant difference as monotherapy [109].

## Data Availability

No new data were created or analyzed in this study. Data sharing is not applicable to this article.

## Abbreviations

The following abbreviations are used in this manuscript:

AI	Artificial intelligence
AE	Aerobic exercise
AMPK	AMP-activated protein kinase
AWGS	Asian Working Group for Sarcopenia
BCAA	Branched-chain amino acid
BIA	Bioelectrical impedance analysis
CT	Computed tomography
DEXA	Dual-energy X-ray absorptiometry
ECM	Extracellular matrix
EWGSOP2	European Working Group on Sarcopenia In Older People 2
FGF	Fibroblast growth factor
GLIS	Global Leadership Initiative on Sarcopenia
HGF	Hepatocyte growth factor
HMB	β-Hydroxy-β-methylbutyrate
IGF-1	Insulin-like growth factor 1
IMAT	Intramuscular adipose tissue
IL	Interleukin
IRS-1	Insulin receptor substrate-1
JAK-STAT3	Janus kinase-signal transducer and activator of transcription 3
mTORC1	Mammalian target of rapamycin complex 1
MuRF1	Muscle RING finger 1
MuSC	Muscle satellite cell
NAD+	Nicotinamide adenine dinucleotide
NFκB	Nuclear factor kappa-light-chain-enhancer of activate B cells
NMJ	Neuromuscular junction
NR	Nicotinamide riboside
NMN	Nicotinamide mononucleotide
PCP	Planar cell polarity
PCr	Phosphocreatine
PGC-1α	Peroxisome proliferator-activated receptor gamma coactivator 1-alpha
PI3K	Phosphoinositide 3-kinase
RAAS	Renin–angiotensin–aldosterone system
ROS	Reactive oxygen species
SARMs	Selective androgen receptor modulators
SCFA	Short-chain fatty acids
SMAD	Mothers against decapentaplegic homolog
SIRT1	Sirtuin 1
TGFβ	Transforming growth factor beta
TNFα	Tumor necrosis factor alpha
Treg	Regulatory T cells
